# High Expression of Solute Carrier Family 2 Member 1 (SLC2A1) in Cancer Cells Is an Independent Unfavorable Prognostic Factor in Pediatric Malignant Peripheral Nerve Sheath Tumor

**DOI:** 10.3390/diagnostics11040598

**Published:** 2021-03-26

**Authors:** Malgorzata A. Krawczyk, Michal Kunc, Malgorzata Styczewska, Anna Gabrych, Gabrielle Karpinsky, Ewa Izycka-Swieszewska, Ewa Bien

**Affiliations:** 1 Department of Pediatrics, Hematology and Oncology, Medical University of Gdansk, 7 Debinki Street, 80-211 Gdansk, Poland; mkrawczyk@gumed.edu.pl (M.A.K.); anna.gabrych@gumed.edu.pl (A.G.); 2 Department of Pathomorphology, Medical University of Gdansk, 17 Smoluchowskiego Street, 80-214 Gdansk, Poland; 3 The English Division Pediatric Oncology Scientific Circle, Medical University of Gdansk, 7 Debinki Street, 80-211 Gdansk, Poland; mstyczewska@gumed.edu.pl; 4 Division of Critical Care Medicine, Department of Pediatrics, University of Colorado School of Medicine, Anschutz Medical Campus, Aurora, CO 80045, USA; gabrielle.karpinsky@childrenscolorado.org; 5 Department of Pathology and Neuropathology, Medical University of Gdansk, 1 Debinki Street, 80-211 Gdansk, Poland; ewa.izycka-swieszewska@gumed.edu.pl

**Keywords:** malignant peripheral nerve sheath tumor, markers of tumor hypoxia, hypoxia-inducible factor-1α, solute carrier family 2 member 1, carbonic anhydrase 9, vascular endothelial growth factor, markers of systemic inflammation, platelet-to-lymphocyte ratio, neutrophil-to-lymphocyte ratio, lymphocyte-to-monocyte ratio

## Abstract

Malignant peripheral nerve sheath tumor (MPNST) in children is a rare mesenchymal malignancy developing predominantly in the setting of neurofibromatosis type 1. The prognosis in advanced MPNST is poor therefore new prognostic markers are highly needed for optimal therapeutic decisions. In many solid tumors, the bidirectional interactions between hypoxia and inflammation in the tumor microenvironment via functions of tumor-associated cells, like neutrophils, lymphocytes and macrophages, have been investigated recently. There is no data whether in MPNST hypoxic microenvironment may translate into systemic inflammation, which is a well-established factor for worse prognosis in cancer patients. Therefore, we investigated the prognostic significance of markers of tumor hypoxia and systemic inflammation in 26 pediatric malignant peripheral nerve sheath tumors (MPNST). Tumor tissue microarrays were stained for hypoxia-inducible factor-1α (HIF1A), solute carrier family 2 member 1 (SLC2A1, also known as glucose transporter 1 (GLUT1)), carbonic anhydrase 9 (CA9), and vascular endothelial growth factor A (VEGFA) and classified into low- or high-expression groups. Baseline complete blood counts and C-reactive protein (CRP) levels were collected for all cases. Neutrophil-to-lymphocyte ratio (NLR), platelet-to-lymphocyte ratio (PLR), and lymphocyte-to-monocyte ratio (LMR) were calculated from age-adjusted complete blood count parameters. Both 10-year RFS and OS were significantly lower in patients with high NLR values (17% vs. 75%, *p* = 0.009, *q* = 0.018; and 31% vs. 100%, *p* = 0.0077, *q* = 0.014; respectively). Ten-year-OS was significantly lower in patients with high expression of SLC2A1 (20.00% vs. 94%, *p* < 0.001, log-rank), high expression of HIF1A (23% vs. 79%, *p* = 0.016, log-rank), and CRP higher than 31 mg/L (11% vs. 82%, *p* = 0.003, *q* = 0.009). Cox’s proportional hazard regression analysis revealed that high expression of SLC2A1 (HR = 3.31, 95% CI = 1.08–10.09, *p* = 0.036) and VEGFA (HR = 4.40, 95% CI = 0.95–20.34, *p* = 0.058) were the independent factors predicting relapse, whereas high SLC2A1 was identified as the independent risk factor for death (HR = 12.20, 95% CI = 2.55–58.33, *p* = 0.002). Patients with high expression of hypoxic markers and low or high NLR/CRP values had the highest events rate, patients with low hypoxic markers and high NLR/CRP had intermediate events rate, while patients with low hypoxic markers and low NLR/CRP had the lowest events rate. SLC2A1 and VEGFA are promising novel prognostic factors in pediatric MPNST. Correlations between hypoxic and systemic inflammatory markers suggest the interplay between local tumor hypoxia and systemic inflammation.

## 1. Introduction

Malignant peripheral nerve sheath tumors (MPNST) are aggressive mesenchymal malignancies affecting mostly adults; however, approximately 14% of cases occur in children. Almost half of pediatric MPNSTs develop in patients with neurofibromatosis type 1 (NF1). The prognosis in MPNST in children has improved in recent decades, with 5-year overall survival (OS) currently reaching 60% [1,2]. The best outcomes are achieved in patients with localized tumors, feasible for complete surgical resection (with negative margins) [1]. Unresectable and/or metastatic MPNST is associated with a poor prognosis, despite multimodal treatment, including chemotherapy and radiation therapy.

In recent years, a few clinical prognostic factors have been investigated in pediatric MPNST, such as the patient’s age, NF1, and stage [3]. There have also been various biomarkers investigated to predict response to chemotherapy (CHT) and survival. In our previous study, we reported that high tumor expressions of cyclin D1, p53, and osteopontin, assessed by immunohistochemistry (IHC), were associated with poor outcomes in children with MPNST [4].

Tumor hypoxia is a result of inadequate blood and oxygen supply caused by insufficient and pathological tumor vasculature. Hypoxia, via the induction of an α subunit of hypoxia-inducible factor-1 (HIF1A), triggers a sequence of events promoting the evolution of aggressive clones from heterogeneous tumor cells [5]. HIF1 activates numerous downstream genes and proteins, including vascular endothelial growth factor A (VEGFA), which promotes neo-angiogenesis [6]**,** solute carrier family 2 member 1 (SLC2A1, also known as glucose transporter 1 (GLUT1)), and enzymes involved in glycolysis, which promote a shift from aerobic to anaerobic metabolic pathways [7,8,9]. These phenomena lead to intracellular acidosis, which may be neutralized, among others, by the upregulation of another HIF1 effector, carbonic anhydrase 9 (CA9) [10]. Thus, not only HIF1A, but also VEGFA, SLC2A1, and CA9 allow the tumor cells to survive in hypoxic environments [11] and are well-established markers of cellular hypoxia [6,10,12].

Interestingly, there are bidirectional interactions between hypoxia and inflammation in the tumor microenvironment. On the one hand, tumor-associated neutrophils (TANs) may contribute to local hypoxia via respiratory burst-dependent oxygen expenditure; on the other hand, HIF1 regulates neutrophil functions and may promote neutrophil expression of pro-tumor factors, such as TNF-alpha, VEGFA, and MMP9 [13]. Other tumor-infiltrating cells, like lymphocytes and macrophages, are also influenced by hypoxia [14]. A hypoxic microenvironment may translate into tumor necrosis and an inflammatory response, eventually leading to systemic inflammation, which is a well-established factor for worse prognosis in cancer patients [15,16].

The extent of systemic inflammatory response may be evaluated by multiple laboratory indices, including the neutrophil-to-lymphocyte ratio (NLR), platelets-to-lymphocyte ratio (PLR), and lymphocyte-to-monocyte (LMR) ratio derived from complete blood count (CBC). As cheap and widely available measures of systemic inflammation, NLR, PLR, and LMR have been well-established as prognostic factors in multiple human malignancies, including pediatric tumors [17,18,19,20]. Serum lactate dehydrogenase (LDH) has been shown to be elevated in many fast-proliferating pediatric tumors, including sarcomas, and may also be associated with the expression of hypoxia markers [21]. Importantly, no study to date has investigated the impact of mediators related to hypoxia on pediatric MPNST prognosis coupled with systemic inflammatory markers and the systemic marker of hypoxia, LDH.

Thus, the aim of the current study is to assess the prognostic significance of HIF1A and other markers of hypoxia expressed by tumor cells and systemic inflammatory markers derived from CBC in children with MPNST. The secondary objective is to investigate the relationships between tumor hypoxia and systemic inflammation in our cohort of patients with MPNST with long-term follow-up.

## 2. Materials and Methods

### 2.1. Patients

Twenty-six pediatric patients (<21 years old) diagnosed with MPNST in Polish pediatric oncology centers and registered in the Polish Pediatric Soft Tissue Sarcomas Registry between March 1992 and November 2013 were enrolled in this study. Medical records of all patients were anonymized, and the following parameters were collected: age, gender, tumor size and location, presence of nodal and distant organ metastases, the Intergroup Rhabdomyosarcoma Study (IRS) stage, response to neoadjuvant CHT, and diagnosis of NF1 according to specific criteria created by the National Institutes of Health. Moreover, the CBC, LDH, and C-reactive protein (CRP) at the time of diagnosis were retrieved. All patients were treated according to Cooperative Weichteilsarkom Study Group (CWS) protocols. The detailed information about patients has been shown in our previous publication [4].

### 2.2. Immunohistochemistry

The tissue microarrays (TMAs) containing representative cores of tumor tissue were constructed as described in our previous study [4]. Subsequently, TMAs were stained with the following antibodies: HIF1A (1:500, Abcam, Cambridge, United Kingdom, code ab463); SLC2A1 (1:200, DAKO, Gdynia, Poland, code A3536); CA9 (1:1000, Abcam, Cambridge, United Kingdom, code ab15086); VEGFA (1:250, Abcam, Cambridge, United Kingdom, code m68334), and assessed semi-quantitatively. Evaluation of hypoxia-markers expression employed estimation of the percentage of positively staining cells (0–5%, 6–25%, 26–50%, more than 50%) and the intensity of staining (no staining, low intensity, intermediate intensity, high intensity). Combined intensity and percentage of immunopositive cells enabled the calculation of the expression coefficient and classification of each patient into a low-expression (score 0–7) or high-expression (score 8–12) group, as described previously and presented in Table 1 [22]. The stainings were examined under light microscopy by two independent pathologists blinded to the clinical data. The percentage of positive cells was estimated manually. In discrepant cases, the final scores were reconciled following discussion.

### 2.3. Markers of Systemic Inflammation

The absolute counts of neutrophils, lymphocytes, monocytes, and platelets at the time of diagnosis were obtained and subsequently divided by the upper cut-off values (neutrophils, monocytes, platelets) or the lower cut-off values (lymphocytes) of the norm according to the NHS Children’s Reference Ranges for Routine Hematology Tests. Afterward, such age-adjusted parameters were used to calculate the neutrophil-to-lymphocyte ratio (NLR), platelet-to-lymphocyte ratio (PLR), and lymphocyte-to-monocyte ratio (LMR). This enabled us to avoid the influence of physiological changes in values of leucocytes’ subsets and platelets on NLR, PLR, and LMR occurring during aging. The characteristics of the study group, including levels of markers of systemic inflammation, immunohistochemical markers, and main clinicopathological features, are presented in Supplementary Material Table S1.

### 2.4. Statistical Analysis

All statistical analyses were performed with the use of Statistica 13 (TIBCO Software Inc, Palo Alto, Santa Clara, CA, USA) licensed to the Medical University of Gdansk. Associations between categorical variables were examined using the two-tailed Fisher’s exact test. Correlations between continuous variables were evaluated with the Spearman correlation coefficient, the Mann–Whitney U test, or the Kruskal–Wallis test. Boxplots were plotted using the “ggplot2” package in R [23,24]. Receiver operating curves (ROC) using the maximal Youden’s index were employed to determine the optimal cut-off values of NLR, PLR, LMR, CRP, and LDH for survival analysis. Kaplan–Meier curves were plotted, and log-rank tests were used to compare survival outcomes between patients with different values of examined parameters. Univariate and multivariable Cox regression models were applied to evaluate the association of clinicopathological variables, hypoxic markers, systemic inflammatory markers status, and survival. Relapse-free survival (RFS) was defined as the period from the time of diagnosis to the date of relapse, whereas overall survival (OS) was calculated from the time of diagnosis to the date of death from any cause.

Obtained *p*-values were controlled for false discovery rate (FDR) with a Benjamini–Hochberg approach. The FDR cut-off was established at 0.05, and adjusted *q*-values are presented together with *p*-values.

## 3. Results

### 3.1. Expression of Hypoxic Markers by MPNST

**Figure 1:**
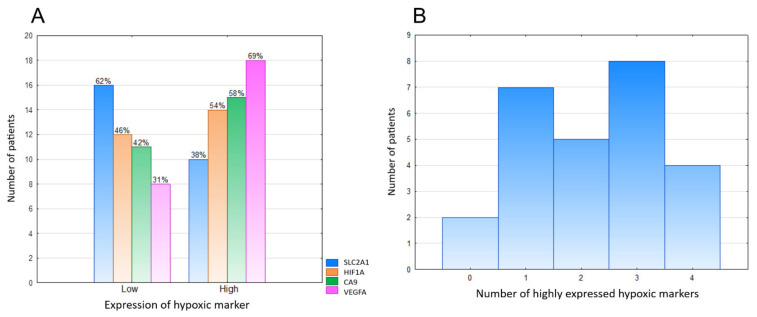
Immunohistochemical expression profile of hypoxic markers (SLC2A1, HIF1A, CA9, VEGFA) in the current cohort of MPNST. VEGFA was the most commonly highly expressed hypoxic marker, whereas SLC2A1 expression was low in most tumors (**A**). The vast majority of MPNSTs highly expressed at least one marker of hypoxia (**B**). Abbreviations: MPNST—malignant peripheral nerve sheath tumor, SLC2A1—solute carrier family 2 member 1, HIF1A—hypoxia-inducible factor-1α, CA9—carbonic anhydrase 9, VEGFA—vascular endothelial growth factor A.

**Figure 2:**
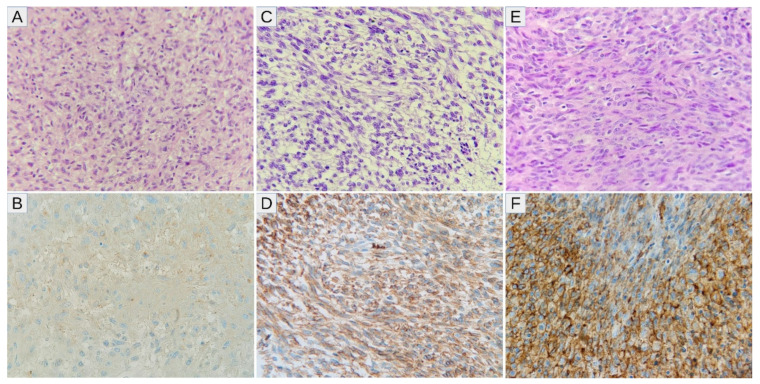
(**A**–**F**) Representative SLC2A1 staining patterns in MPNSTs with the accompanying hematoxylin/eosin images: (**A**,**B**) A case with low-intensity (1+) membrane-cytoplasmic expression of SLC2A1 (the expression coefficient—7, low-expression). (**C**,**D**) Intermediate-intensity (2+) membranous staining intensity; (the expression coefficient—11, high-expression). (**E**,**F**) Predominantly strong (3+) to intermediate (2+) intensity membranous immunoreaction with SLC2A1 (the expression coefficient—12, high-expression). Abbreviations: SLC2A1—solute carrier family 2 member 1, MPNST—malignant peripheral nerve sheath tumor.

**Figure 3:**
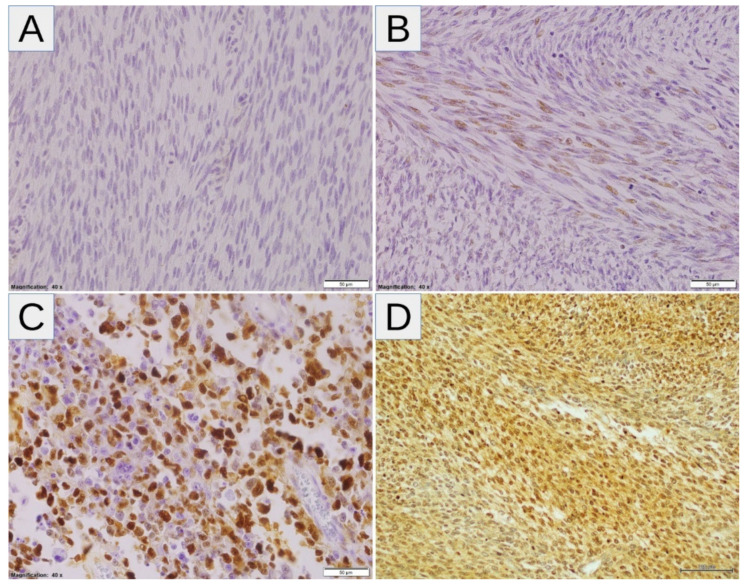
(**A**–**D**) Representative HIF1A staining patterns in MPNST: (**A**,**B**) Cases with absent (**A**) and low-intensity (1+) (**B**) nuclear expression of HIF1A (the expression coefficient—0, low-expression and 4, low-expression, respectively). (**C**,**D**) Cases with predominantly high-intensity (3+) nuclear (**C**) and nuclear/cytoplasmic (**D**) expression of HIF1A (the expression coefficient—12, high-expression in both cases). Abbreviations: HIF1A—hypoxia-inducible factor-1α, MPNST—malignant peripheral nerve sheath tumor.

**Figure 4:**
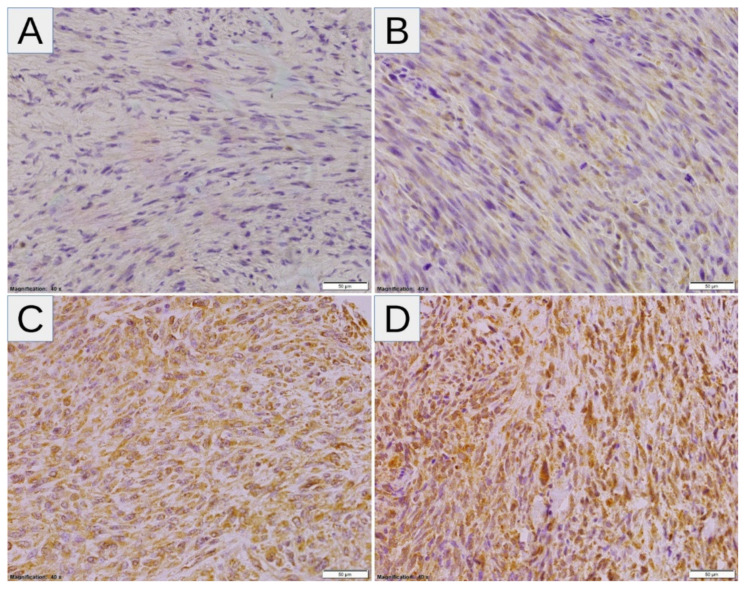
(**A**–**D**) Representative VEGFA staining patterns in MPNST: (**A**,**B**) Cases with absent-to-very-low (**A**) and low-to-intermediate (1+/2+) (**B**) cytoplasmic expression of VEGFA (the expression coefficient—1, low-expression and 7, low-expression, respectively). (**C**,**D**) Cases with predominantly intermediate/high- (2+/3+) (**C**) and high-intensity (3+) (**D**) cytoplasmic expression of VEGFA (the expression coefficient—11, high-expression, and 12, high-expression, respectively). Abbreviations: VEGFA—vascular endothelial growth factor A, MPNST—malignant peripheral nerve sheath tumor.

**Figure 5:**
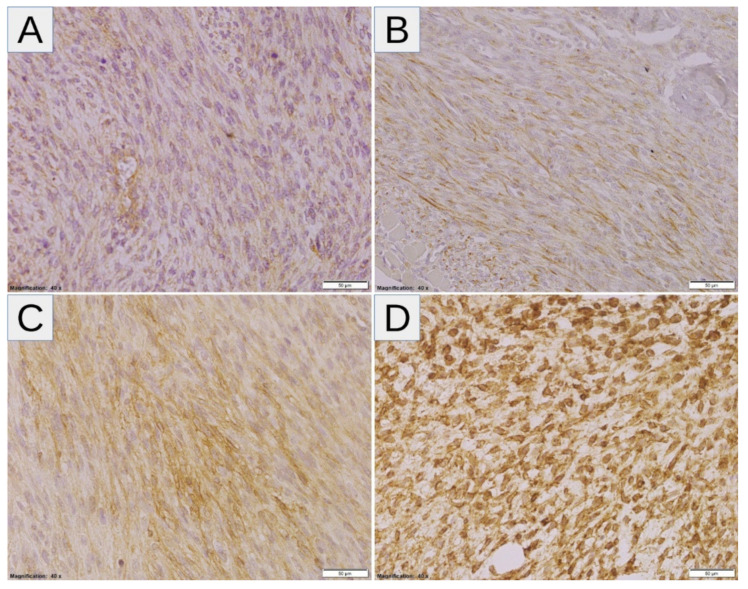
(**A**–**D**) Representative CA9 staining patterns in MPNST: (**A**,**B**) Cases with low- (1+) (**A**) and intermediate-intensity (2+) (**B**) predominantly membranous expression of CA9 (the expression coefficient—7, low-expression and 8, high-expression, respectively). (**C**,**D**) Cases with predominantly intermediate/high (2+/3+) (**C**) and high intensity (3+) (**D**) membranous and cytoplasmic expression of CA9 (the expression coefficient—11, high-expression, and 12, high-expression, respectively). Abbreviations: CA9—carbonic anhydrase 9, MPNST—malignant peripheral nerve sheath tumor.

High expressions of SLC2A1, HIF1A, VEGFA, and CA9 were noted in 10/26 (38.46%), 14/26 (53.85%), 18/26 (69.23%), and 15/26 (57.69%) samples, respectively (Figure 1A). Twenty-four tumors (92.31%) displayed high levels of at least one marker of hypoxia, whereas two tumors (7.69%) showed consistent low expression of all markers (Figure 1B). After correction for multiple comparisons, no significant relationships between hypoxic markers and clinicopathological variables were noted (Table 2). There was a trend towards a positive correlation between SLC2A1 and CA9 (*p* = 0.014, *q* = 0.084; Fisher’s exact test), and no other associations were observed between hypoxic markers (Table 3). The representative examples of the staining patterns of analyzed markers in MPNST tumors samples are shown in Figure 2 (SLC2A1), Figure 3 (HIF1A), Figure 4 (VEGFA), and Figure 5 (CA9).

### 3.2. Complete Blood Count, LDH, and CRP

**Figure 6:**
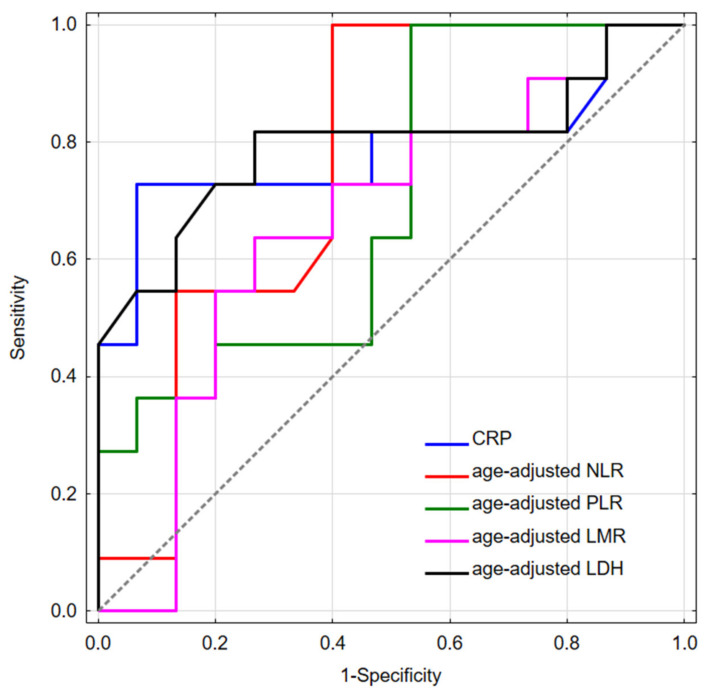
Receiver operating curves plotted for age-adjusted NLR, PLR, LMR, and LDH in pediatric patients with MPNST to predict events. Abbreviations: CRP—C-reactive protein, NLR—neutrophil-to-lymphocyte ratio, PLR—platelet-to-lymphocyte ratio, LMR—lymphocyte-to-monocyte ratio, LDH—lactate dehydrogenase.

Receiver operating curves plotted for NLR, PLR, LMR, LDH, and CRP are shown in Figure 6. Table 4 presents AUC with confidence intervals for each marker. Established cut-off values were 0.22, 0.45, 3.88, and 0.99, respectively. CRP was not age-adjusted, and its cut-off value was 31. Due to the unsatisfactory discriminating value of PLR, LMR, and LDH, only NLR and CRP were included in the further analyzes.

**Figure 7:**
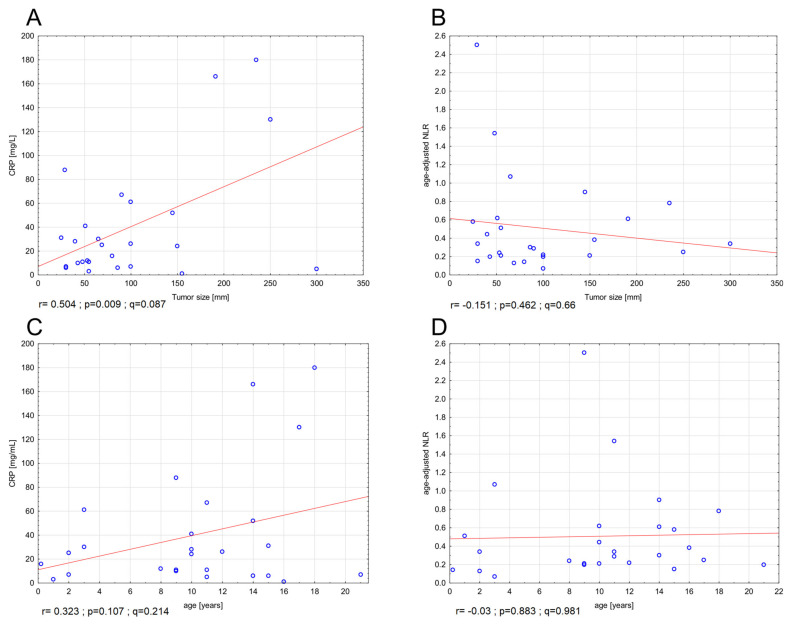
Correlations between CRP and tumor size (**A**); NLR and tumor size (**B**); CRP and age (**C**); NLR and age (**D**). Abbreviations: CRP—C-reactive protein, NLR—neutrophil-to-lymphocyte ratio.

**Figure 8:**
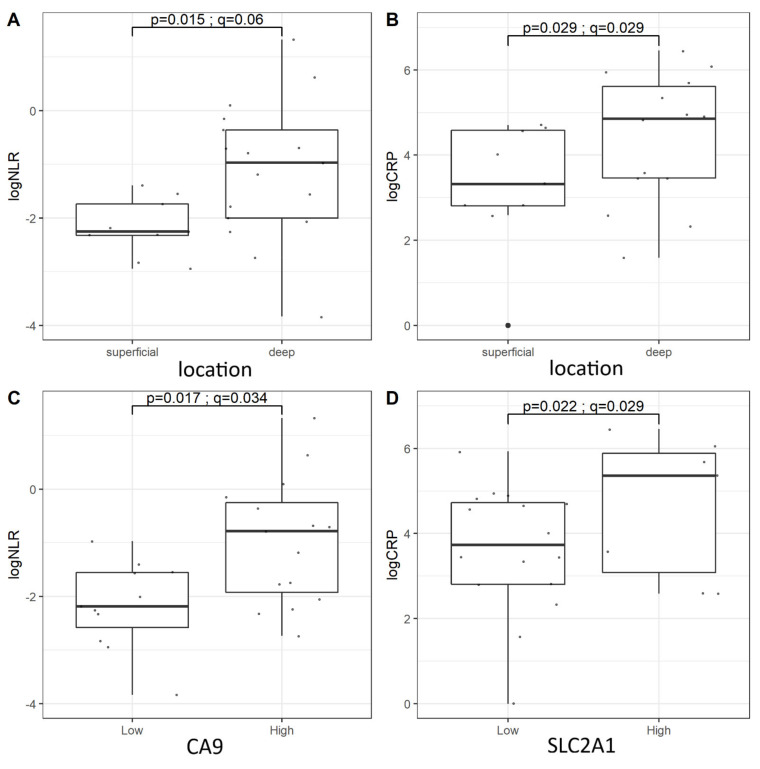
The associations between age-adjusted NLR and CRP levels and tumor location (**A**,**B**); and hypoxic markers (CA9, SLC2A1) expressions (**C**,**D**). Abbreviations: NLR—neutrophil-to-lymphocyte ratio, CRP—C-reactive protein, CA9—carbonic anhydrase 9, SLC2A1—solute carrier family 2 member 1.

There was a trend toward higher CRP levels in larger tumors (r = 0.5036; *p* = 0.009, *q* = 0.087, Spearman’s rho) (Figure 7A), whereas NLR levels were unrelated to tumor size (Figure 7B). CRP levels tended to be higher in older patients, but this finding was statistically insignificant (Figure 7C), whereas, as expected, age-adjusted NLR was not correlated with patients’ age (Figure 7D). Patients with deep-seated tumors tended to have higher levels of both NLR and CRP. Moreover, we observed a relationship between markers of hypoxia, CA9 and SLC2A1, with NLR and CRP, respectively [Figure 8].

### 3.3. Survival Analysis—Markers of Hypoxia

**Figure 9:**
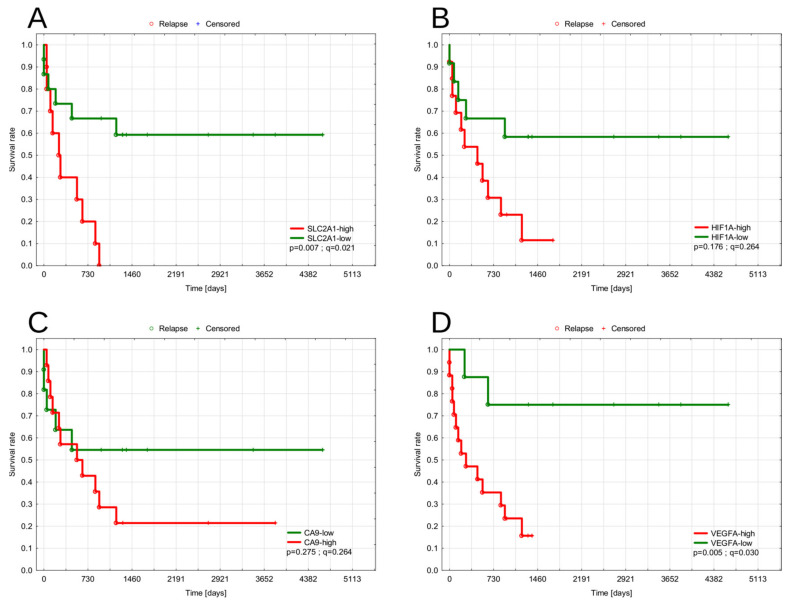

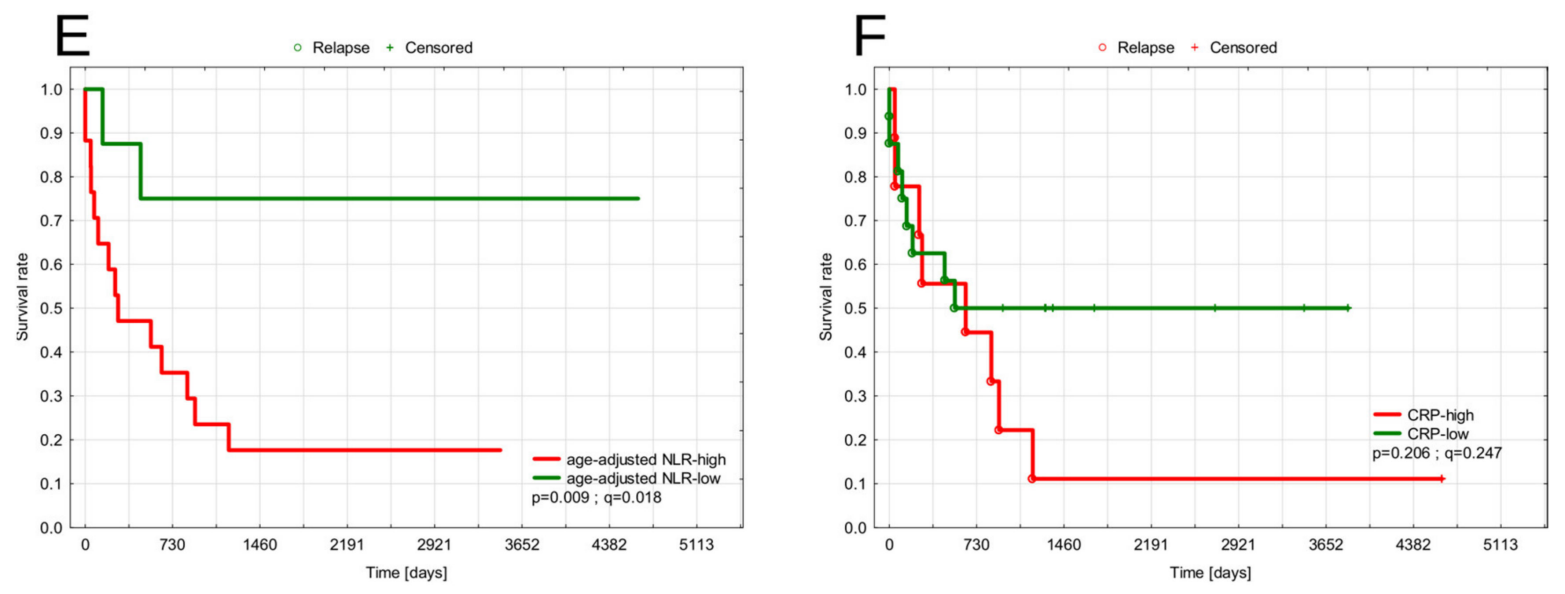
Kaplan–Meier curves showing the probability of relapse-free survival grouped by SLC2A1 (**A**), HIF-1a (**B**), CA9 (**C**), VEGFA (**D**), NLR (**E**), and CRP (**F**). Abbreviations: SLC2A1—solute carrier family 2 member 1, HIF1A—hypoxia-inducible factor-1α, CA9—carbonic anhydrase 9, VEGFA—vascular endothelial growth factor A, NLR—neutrophil-to-lymphocyte ratio, CRP—C-reactive protein.

**Figure 10:**
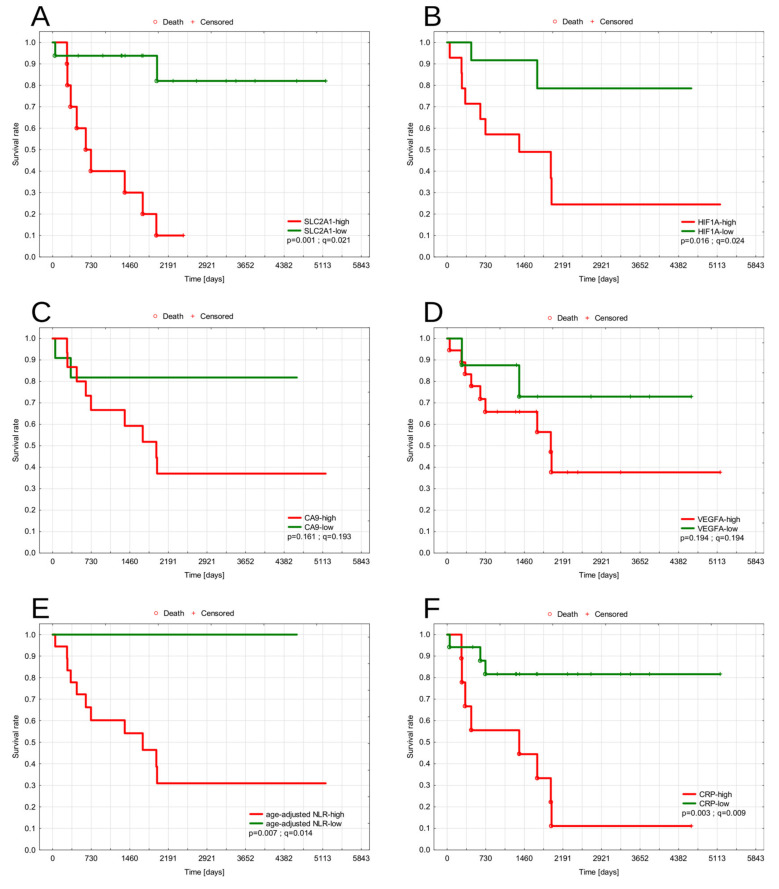
Kaplan–Meier plots showing overall survival stratified by SLC2A1 (**A**), HIF1A (**B**), CA9 (**C**), VEGFA (**D**), NLR (**E**), CRP (**F**). Abbreviations: SLC2A1—solute carrier family 2 member 1, HIF1A—hypoxia-inducible factor-1α, CA9—carbonic anhydrase 9, VEGFA—vascular endothelial growth factor A, NLR—neutrophil-to-lymphocyte ratio, CRP—C-reactive protein.

We observed a general trend for worse RFS in MPNSTs characterized by greater expression of hypoxic markers. Most of the patients relapsed within the first two years of follow-up. The highest risk of early relapse was seen in tumors with high SLC2A1 and high VEGFA expression (Table 5). Specifically, in our cohort, every case of MPNST with high SLC2A1 expression relapsed within three years after diagnosis. Similar findings were observed when analyzing OS. Ten-year-OS was significantly lower in patients with high expression of SLC2A1 (20% vs. 94%, *p* < 0.001, *q* = 0.036, log-rank), and high expression of HIF1A (23% vs. 79%, *p* = 0.016, *q* = 0.024, log-rank) (Table 5). Kaplan–Meier curves grouped by investigated markers are shown in Figure 9 and Figure 10.

### 3.4. Survival Analysis—NLR and CRP

Elevated levels of markers of systemic inflammation were found to be associated with worse outcomes in our cohort. Both 10-year RFS and OS were significantly lower in patients with high NLR value (17% vs. 75%, *p* = 0.009, *q* = 0.018; and 31% vs. 100%, *p* = 0.0077, *q* = 0.014; respectively). CRP higher than 31 mg/L was significantly associated with poor 10-year OS (11% vs. 82%, *p* = 0.003, *q* = 0.009).

To further investigate the association between investigated markers and survival, we plotted Kaplan–Meier curves for combined hypoxic markers and systemic inflammatory markers (Supplementary Figure S1). To perform this analysis, we chose SLC2A1, HIF1A, VEGFA, NLR, and CRP, the markers which significantly affect survival rates. We observed substratification of patients into three groups in terms of outcomes. Patients with low levels of both markers of hypoxia and systemic inflammation were characterized by better survival. On the contrary, MPNST cases with high expression of hypoxic markers and elevated CRP or NLR had the worst outcomes. Finally, in the group of patients with low levels of hypoxic markers and increased systemic inflammatory markers, the risk of relapse or death was intermediate. These associations were most prominent for the combination of SLC2A1/NLR and HIF1A/NLR.

### 3.5. Multivariable Model

Cox’s proportional hazard regression analysis was used to determine if hypoxic markers or NLR and CRP independently influence OS and RFS in pediatric MPNST patients. The model was adjusted to the stage, tumor location (superficial/deep), the presence of distal and/or nodal metastases, and diagnosis of NF1. The analysis revealed that high-expression of SLC2A1 (HR = 3.31, 95% CI = 1.08–10.09, *p* = 0.036) and VEGFA (HR = 4.40, 95% CI = 0.95–20.34, *p* = 0.058) were the independent factors predicting relapse, whereas high SLC2A1 was identified as the independent risk factor for death (HR = 12.20, 95% CI = 2.55–58.33, *p* = 0.002).

## 4. Discussion

MPNST is an uncommon malignancy which is associated with substantial mortality in pediatric patients. Hence, novel prognostic and predictive factors are being investigated. HIF1A has been shown to be overproduced by MPNST cells even in a normoxic environment, and its silencing or inhibition leads to failure of growth and apoptosis of MPNST cell lines [25]. The study of Rad et al. demonstrated that knockdown of STAT3 in MPNST cell lines leads to silencing of the HIF1/VEGF signaling axis. This consequently inhibits MPNST cells migration, invasion, and tumor formation [26]. Accordingly, HIF1A has recently been shown to be a poor prognostic factor in MPNST in adults [23].

Our study supports the abovementioned results and provides novel data on prognostic significance of hypoxic markers in MPNST. Particularly, our findings suggest a strong association of high SLC2A1 expression with inferior outcomes in MPNST patients. SLC2A1 expression is promoted in hypoxia, providing glucose influx to maintain anaerobic glycolysis, contributing to the Warburg effect. Multiple cancer-related pathways up-regulate SLC2A1 in tumor cells, including HIF1, MYC, PI3K-Akt, and RAS-MAPK [9]. Inhibition of HIF1A by chetomin decreases the expression of downstream gene-encoding of SLC2A1 in MPNST cell lines [25]. Corresponding to our study, high expression of SLC2A1 has been found to be associated with inferior outcomes in various malignancies in adults, including pancreatic cancer, colorectal cancer, breast cancer, lung cancer, and others [27,28,29,30]. Accordingly, pediatric cancers overexpressing SLC2A1, like adrenocortical carcinomas and liver vascular tumors, have worse prognosis than SLC2A1-negative tumors [31,32]. In patients with Wilms’ tumor and neuroblastoma, high SLC2A1 expression is correlated with unfavorable histology and high-risk features [33,34]. Considering pediatric sarcomas, our team previously reported that abundance of hypoxic markers (specifically SLC2A1 and CA9) in rhabdomyosarcoma characterized aggressive tumors resistant to neoadjuvant chemotherapy [22]. In the current study, we noted a trend towards higher SLC2A1 expression in deeply located tumors in MPNST patients, but it was statistically insignificant. This trend may suggest that deeply located tumors are more likely to develop hypoxia and activate appropriate pathways leading to increased expression of hypoxic markers. Deep location was also correlated with higher levels of systemic inflammatory markers. Finally, SLC2A1 and CA9 expression correlated with CRP and NLR levels, respectively. These results may indicate the interactions between hypoxia and systemic inflammation in patients with MPNST. Targeting SLC2A1 activity seems to be a promising strategy for the treatment of cancer with prominent hypoxic response and Warburg effect; however, utility of SLC2A1 inhibitors in MPNST is yet to be established.

High expression of other markers of hypoxia investigated in the current study was also associated with inferior outcomes, but besides SLC2A1, only VEGFA was incorporated in the multivariable model. VEGF is a crucial trigger to activate an angiogenic switch, where new blood vessels are produced within a tumor, providing oxygen and nutrients to a growing mass [35]. Bevacizumab, a drug inhibiting VEGF, is used to treat patients with some advanced malignancies, like ovarian cancer. Unfortunately, the results of a clinical trial combining everolimus with bevacizumab in the treatment of refractory MPNST showed clinical benefit only in three out of 25 enrolled patients [36]. Another VEGF inhibitor, axitinib, was evaluated in a phase 1 clinical trial enrolling children and adolescents with recurrent/refractory solid tumors. Among two patients with MPNST, one achieved stabilization of their disease [37].

To our knowledge, this is the very first study investigating combined markers of tumor hypoxia and systemic inflammatory markers. The impact of systemic inflammation on the clinical course of pediatric cancers is still sparse, but a few reports have suggested that high PLR, NLR, and LMR are associated with poor outcomes in children with neuroblastoma, Wilms tumor, salivary gland tumors, and some sarcomas [17,18,19,20].

Systemic inflammation may promote cancer progression via cytokines, modifying interactions between neoplastic and non-neoplastic cells, facilitating evasion of the immune response and shifting metabolic pathways [38]. Moreover, systemic inflammation contributes to cachexia and chronic fatigue. The direct mechanism or pathway connecting local tumor necrosis and hypoxia is still unknown; however, multiple indirect proofs suggest such association. For example, in a study by Bousquet et al., a low ratio of reactive oxygen species to mitochondrial DNA, indicating tumor hypoxia, was associated with elevated systemic inflammation factors, such as CRP and interleukin-1 receptor antagonist, in patients with locally advanced rectal cancer [39]. In the current study, we observed a correlation between high levels of inflammatory markers (NLR and CRP) and tumor expression of CA9 and SLC2A1, which further supports the interplay between hypoxia and inflammation in MPNST. The combination of NLR or PLR with expression of selected hypoxic markers substratified the MPNST patients in terms of survival. The patients with neither prominent inflammatory response nor tumor hypoxia were characterized by the best prognosis.

## 5. Conclusions

In the current study, we investigated the clinical significance of tumor expressions of HIF1A, VEGFA, CA9, and SLC2A1 assessed by IHC and markers of systemic inflammation (NLR and CRP) in pediatric patients with MPNST. SLC2A1 and VEGFA were identified as promising novel prognostic factors. Especially IHC for SLC2A1 could be easily implemented in MPNST diagnostics since it is easily accessible and commonly used in pathology laboratories worldwide. Moreover, the results suggest an interplay between local tumor hypoxia and the systemic inflammatory response. Further multicenter studies are necessary to fully assess the role of these markers in MPNST.

## Figures and Tables

**Table 1:** The assessment of the expression coefficient of analyzed markers in neoplastic cells based on the intensity of the immunohistochemical staining and the percentage of the immunopositive cells**.**

		Percentage of Immunopositive Cells			
Intensity of the IHC staining		>50%	26–50%	6–25%	0–5%
	High +++	**12**	**9**	**6**	**3**
	Intermediate ++	**11**	**8**	**5**	**2**
	Low +	**10**	**7**	**4**	**1**
	No staining	**0**			

Abbreviation: IHC—immunohistochemistry.

**Table 2:** Associations between markers of hypoxia and the selected clinicopathological variables. Significant *p*-values after Benjamini–Hochberg correction are written in bold letters.

Variable		SLC2A1			HIF1A			VEGFA			CA9		
		Low	High	*p*-Value/ *q*-Value	Low	High	*p*-Value/ *q*-Value	Low	High	*p*-Value/ *q*-Value	Low	High	*p*-Value/ *q*-Value
Baseline distant and/or nodal metastases	No	13	5	0.189/0.378	11	7	**0.035/0.420**	5	13	0.667/0.889	8	10	1.000/1.000
	Yes	3	5		1	7		3	5		3	5	
Location	Superficial	8	1	0.087/0.552	5	4	0.484/0.830	3	6	1.000/1.000	6	3	0.103/0.412
	Deep	8	9		7	10		5	12		5	12	
NF1	No	12	4	0.108/0.324	8	8	0.619/0.928	5	11	1.000/1.000	9	7	0.109/0.262
	Yes	4	6		4	6		3	7		2	8	

Abbreviations: SLC2A1—solute carrier family 2 member 1, HIF1A—hypoxia-inducible factor-1α, VEGFA—vascular endothelial growth factor A, CA9—carbonic anhydrase 9, NF1—neurofibromatosis type 1.

**Table 3:** Pair-wise correlations between markers of hypoxia. Significant *p*-values after Benjamini–Hochberg correction are written in bold letters.

		SLC2A1			HIF1A			VEGFA		
		Low	High	*p*-Value/ *q*-Value	Low	High	*p*-Value/ *q*-Value	Low	High	*p*-Value/ *q*-Value
HIF1A	Low	9	3	0.191/0.573	N/A			N/A		
	High	7	7							
VEGFA	Low	6	2	0.419/0.628	5	3	0.265/0.530	N/A		
	High	10	8		7	11				
CA9	Low	10	1	**0.014/0.084**	5	6	0.951/1.000	3	8	1.000/1.000
	High	6	9		7	8		5	10	

Abbreviations: SLC2A1—solute carrier family 2 member 1, HIF1A—hypoxia-inducible factor-1α, VEGFA—vascular endothelial growth factor A, CA9—carbonic anhydrase 9, N/A—not applicable.

**Table 4:** Areas under curves and cut-off values (established with Youden index) for investigated markers.

Variable	AUC	±95% CI	Cut-Off	*p*-Value	*q*-Value
NLR	0.76	0.57–0.95	0.24	0.007	0.017
PLR	0.69	0.49–0.90	0.45	0.062	0.103
LMR	0.66	0.44–0.88	3.88	0.149	0.149
CRP	0.78	0.57–0.99	31 mg/L	0.007	0.035
LDH	0.66	0.44–0.88	0.99	0.144	0.180

Abbreviations: NLR—neutrophil-to-lymphocyte ratio, PLR—platelet-to-lymphocyte ratio, LMR—lymphocyte-to-monocyte ratio, CRP—C-reactive protein, LDH—lactate dehydrogenase, AUC—area under curve.

**Table 5:** Comparison of survival between groups with low and high expression of investigated markers.

Variable	2-Year RFS [%]		10-Year RFS [%]		Log-Rank *p*-Value	Log-Rank *q*-Value	Multivariate Cox Regression		
	Low	High	Low	High			HR	95% CI	*p*-Value
SLC2A1	67	20	59	0	0.007	0.021	3.31	1.08–10.09	0.036
HIF1A	67	31	58	11	0.176	0.264	*		
CA9	55	43	55	22	0.275	0.275	*		
VEGFA	75	36	75	16	0.005	0.030	4.40	0.95–20.34	0.058
NLR	75	35	75	17	0.009	0.018	*		
CRP	50	44	50	11	0.206	0.247	*		
	**2-year OS [%]**		**10-year OS [%]**						
SLC2A1	94	40	82	10	<0.001	0.036	12.20	2.55–58.33	0.002
HIF1A	92	57	79	23	0.016	0.024	*		
CA9	82	67	82	37	0.161	0.193	*		
VEGFA	88	66	73	38	0.194	0.194	*		
NLR	100	60	100	31	0.007	0.014	*		
CRP	82	56	82	11	0.003	0.009	*		

Abbreviations: RFS—relapse-free survival; OS—overall survival; HR—hazard ratio; CI—confidence interval, SLC2A1—solute carrier family 2 member 1, HIF1A—hypoxia-inducible factor-1α, CA9—carbonic anhydrase 9, VEGFA—vascular endothelial growth factor A, NLR—neutrophil-to-lymphocyte ratio, CRP—C-reactive protein, *—not included in multivariable model.

## Supplementary Materials

The following are available online at https://www.mdpi.com/article/10.3390/diagnostics11040598/s1. Supplementary Table S1, summary of demographic and clinicopathological characteristics of the study group, including systemic inflammatory markers and the expression of hypoxic markers; Supplementary Figure S1, Kaplan–Meier curves plotted for overall survival (A–D) and relapse-free survival (E,F) for combinations of hypoxic and inflammatory markers.
