# Supplementary material for: High Expression of Solute Carrier Family 2 Member 1 (SLC2A1) in Cancer Cells Is an Independent Unfavorable Prognostic Factor in Pediatric Malignant Peripheral Nerve Sheath Tumor
Source: Diagnostics (Basel). 2021 Mar 26;11(4):598. doi: 10.3390/diagnostics11040598 (PMC8065586; doi:10.3390/diagnostics11040598)
Supplement: Supplementary file 1 [file diagnostics-11-00598-s001.zip › Supplementary_Table_MPNST.docx]

| # | Sex | Age [y] | Tumor location | Stage | Relapse during follow-up | Death during follow up | NLR | Age-adjusted NLR | PLR | Age-adjusted PLR | LMR | Age-adjusted LMR | CRP [mg/L] | LDH [U/L] | Age-adjusted LDH | SLC2A1 | HIF1A | CA9 | VEGFA |
| --- | --- | --- | --- | --- | --- | --- | --- | --- | --- | --- | --- | --- | --- | --- | --- | --- | --- | --- | --- |
| 1 | F | 17 | Chest | II | Yes | Yes | 1,82 | 0,25 ↑ | 164,32 | 0,45 ↑ | 7,30 | 5,31 | 130 | 315 | 1,35 ↑ | H | H | L | H |
| 2 | F | 21 | Limb | III | No | No | 1,48 | 0,20 | 291,84 | 0,80 ↑ | 1,72 | 1,25 ↓ | 7 | 348 | 1,57 ↑ | L | L | H | L |
| 3 | M | 10 | H&N | IV | Yes | Yes | 3,28 | 0,62 ↑ | 161,71 | 0,61 ↑ | 2,49 | 1,66 ↓ | 41 | 204 | 0,7 | H | H | H | H |
| 4 | F | 16 | Limb | III | No | No | 2,79 | 0,38 ↑ | 125,00 | 0,34 | 10,00 | 7,27 | 1 | 462 | 1,98 ↑ | L | L | L | H |
| 5 | F | 1 | Chest | II | Yes | No | 1,08 | 0,51 ↑ | 70,77 | 0,71 ↑ | 28,18 | 8,45 | 3 | 328 | 0,89 | L | L | L | H |
| 6 | F | 0 | Chest | IV | No | No | 0,29 | 0,14 | 27,23 | 0,27 | 105,27 | 31,58 | 16 | 263 | 0,6 | L | H | L | H |
| 7 | M | 3 | Limb | III | No | No | 0,38 | 0,07 | 50,16 | 0,19 | 19,09 | 15,27 | 61 | 238 | 0,64 | L | L | L | L |
| 8 | F | 14 | Limb | III | Yes | Yes | 2,17 | 0,30 ↑ | 185,77 | 0,51 ↑ | 3,29 | 2,39 ↓ | 6 | 479 | 1,69 ↑ | H | H | H | H |
| 9 | M | 18 | Limb | III | Yes | Yes | 5,70 | 0,78 ↑ | 1160,61 | 3,19 ↑ | 2,36 | 1,71 ↓ | 180 | 220 | 0,99 ↑ | H | L | H | H |
| 10 | M | 15 | Limb | II | Yes | Yes | 4,21 | 0,58 ↑ | 1184,21 | 3,26 ↑ | 11,40 | 8,29 | 31 | 278 | 0,98 | L | H | H | H |
| 11 | F | 14 | A/P | IV | Yes | Yes | 4,45 | 0,61 ↑ | 222,31 | 0,61 ↑ | 2,20 | 1,60 ↓ | 166 | 228 | 0,81 | H | H | H | H |
| 12 | M | 11 | A/P | IV | Yes | Yes | 1,81 | 0,34 ↑ | 139,78 | 0,52 ↑ | 5,81 | 3,89 ↓ | 5 | 275 | 0,94 | L | H | L | H |
| 13 | M | 9 | H&N | III | Yes | Yes | 13,34 | 2,50 ↑ | 763,51 | 2,86 ↑ | 10,57 | 7,05 | 87 | 658 | 2,27 ↑ | H | L | H | H |
| 14 | M | 10 | Chest | III | No | No | 2,34 | 0,44 ↑ | 246,82 | 0,93 ↑ | 2,88 | 1,92 ↓ | 28 | 652 | 2,23 ↑ | L | L | H | L |
| 15 | M | 14 | A/P | III | Yes | Yes | 6,55 | 0,90 ↑ | 527,27 | 1,45 ↑ | 1,75 | 1,27 ↓ | 52 | 238 | 0,84 | H | H | H | L |
| 16 | M | 12 | Limb | III | Yes | No | 1,19 | 0,22 ↑ | 227,70 | 0,85 ↑ | 9,59 | 6,39 | 26 | 440 | 1,5 ↑ | L | H | L | H |
| 17 | M | 10 | Limb | II | No | No | 1,10 | 0,21 | 179,76 | 0,67 ↑ | 5,74 | 3,83 ↓ | 24 | 349 | 1,19 ↑ | L | L | H | L |
| 18 | F | 9 | Chest | III | No | No | 1,14 | 0,21 | 57,14 | 0,21 | 8,29 | 5,53 ↓ | 11 | 418 | 1,44 ↑ | L | H | L | L |
| 19 | F | 11 | H&N | III | Yes | No | 8,22 | 1,54 ↑ | 546,88 | 2,05 ↑ | 1,78 | 1,19 ↓ | 11 | 278 | 0,95 | L | L | H | H |
| 20 | F | 3 | A/P | III | Yes | No | 6,09 | 1,07 ↑ | 262,73 | 0,99 ↑ | 5,50 | 4,40 | 30 | 285 | 0,77 | L | H | H | H |
| 21 | M | 15 | H&N | III | Yes | No | 1,07 | 0,15 | 74,07 | 0,20 | 6,94 | 5,05 | 6 | 827 | 2,92 | H | L | H | H |
| 22 | F | 8 | H&N | III | Yes | Yes | 1,30 | 0,24 ↑ | 141,25 | 0,53 ↑ | 21,05 | 14,04 | 12 | 159 | 0,55 | H | H | H | H |
| 23 | M | 11 | Limb | IV | Yes | Yes | 1,55 | 0,29 ↑ | 233,33 | 0,88 ↑ | 4,82 | 3,21 ↓ | 67 | 199 | 0,68 | H | H | H | L |
| 24 | M | 9 | Limb | III | Yes | No | 1,04 | 0,20 | 85,11 | 0,32 | 12,16 | 8,11 | 10 | 344 | 1,19 ↑ | L | L | L | H |
| 25 | M | 2 | Limb | III | No | No | 0,73 | 0,13 | 107,29 | 0,40 | 9,52 | 7,61 | 25 | 385 | 1,04 ↑ | L | L | L | H |
| 26 | F | 2 | A/P | II | No | No | 1,91 | 0,34 ↑ | 147,02 | 0,55 ↑ | 12,08 | 9,66 | 7 | 1137 | 3,07 ↑ | L | L | L | L |
|  | | | | | |  | | | | | | | | | |  | | | |
|  |  |  |  |  |  | Mean | 2.96 | 0.51 | 283.98 | 0.94 | 11.99 | 6.46 | 40.14 | 384.88 | 1.29 |  |  |  |  |
|  |  |  |  |  |  | Median | 1.81 | 0.32 | 172.03 | 0.61 | 7.12 | 5.18 | 24.5 | 321.25 | 1.01 |  |  |  |  |

Supplementary Table. Summary of demographic and clinicopathological characteristics of the study group, including systemic inflammatory markers and expression of hypoxic markers. Marks: ↑/↓ = higher/lower than calculated cut-off value,

Abbreviations: y – years; NLR – neutrophil-to-lymphocyte ratio; PLR – platelet-to-lymphocyte ratio; LMR – lymphocyte-to-monocyte ratio; CRP – C-reactive protein; LDH – lactate dehydrogenase; SLC2A1 - Solute carrier family 2 member 1; HIF1A – Hypoxia-inducible factor 1; CA9 – Carbonic Anhydrase 9; VEGFA – Vascular endothelial growth factor A; F – female; M – male; H&N – head and neck; A/P – abdomen/pelvis
