# Supplementary figures and images for: High Expression of Solute Carrier Family 2 Member 1 (SLC2A1) in Cancer Cells Is an Independent Unfavorable Prognostic Factor in Pediatric Malignant Peripheral Nerve Sheath Tumor
Source: Diagnostics (Basel). 2021 Mar 26;11(4):598. doi: 10.3390/diagnostics11040598 (PMC8065586; doi:10.3390/diagnostics11040598)

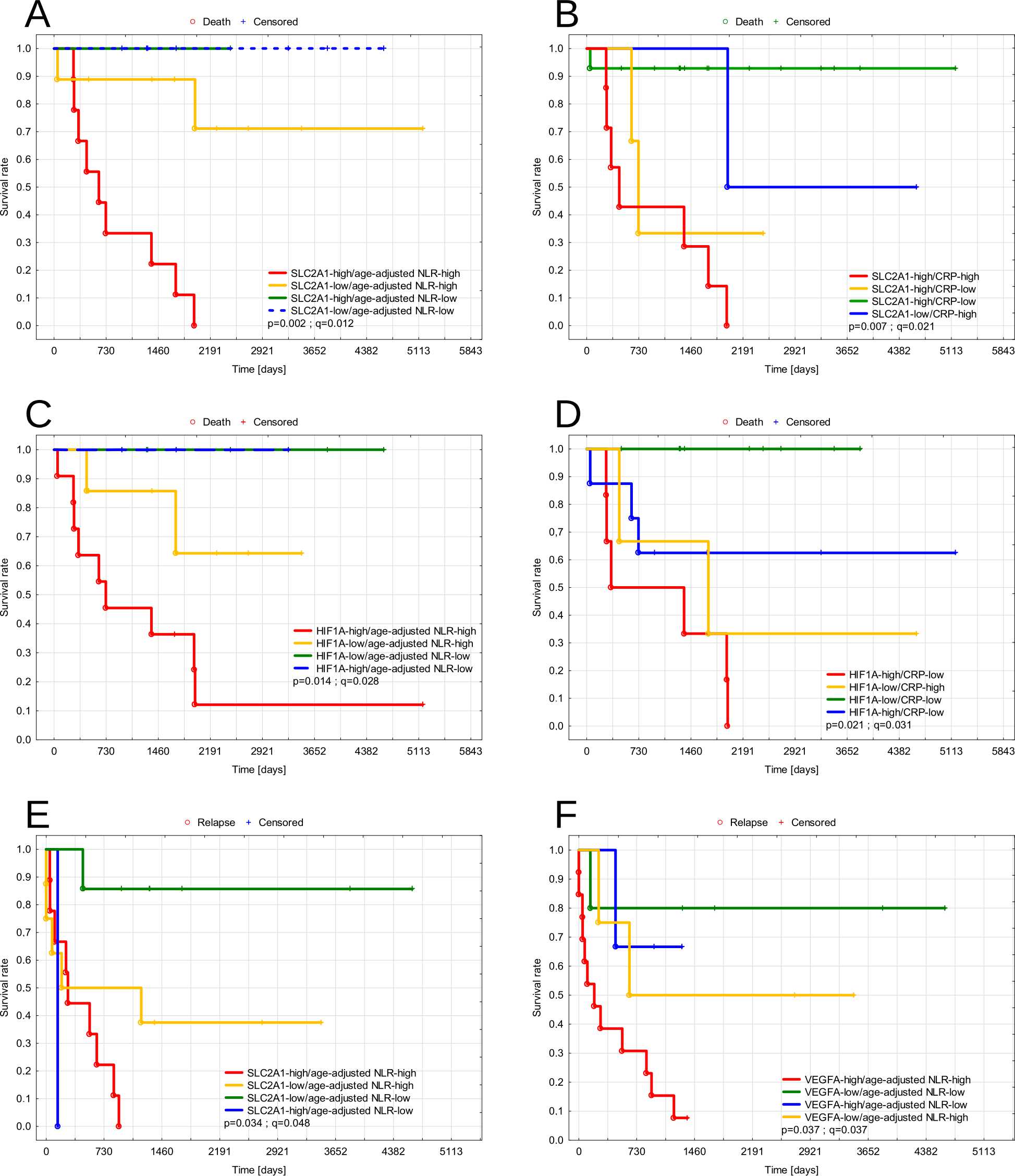

Supplement: Supplementary file 1 [file diagnostics-11-00598-s001.zip › SupFig1.tif]
